# Regulation of Intrinsic and Extrinsic Apoptotic Pathways in Osteosarcoma Cells Following Oleandrin Treatment

**DOI:** 10.3390/ijms17111950

**Published:** 2016-11-23

**Authors:** Yunlong Ma, Bin Zhu, Lei Yong, Chunyu Song, Xiao Liu, Huilei Yu, Peng Wang, Zhongjun Liu, Xiaoguang Liu

**Affiliations:** 1 Department of Orthopedics, Peking University Third Hospital, No. 49, North Garden Street, Haidian District, Beijing 100191, China; mayl_pth2012@163.com (Y.M.); eathonecho@163.com (L.Y.); lx86831@163.com (X.L.); yuhuileiah@126.com (H.Y.); wpspine@sina.com (P.W.); zjlspine@163.com (Z.L.); 2 The Center for Pain Medicine, Peking University Third Hospital, No. 49, North Garden Street, Haidian District, Beijing 100191, China; zhubin_ortho@163.com; 3 Department of Anesthesiology, Peking University Third Hospital, No. 49, North Garden Street, Haidian District, Beijing 100191, China; songchunyu0809@163.com

**Keywords:** oleandrin, osteosarcoma, apoptosis, caspases

## Abstract

Our previous study has reported the anti-tumor effect of oleandrin on osteosarcoma (OS) cells. In the current study, we mainly explored its potential regulation on intrinsic and extrinsic apoptotic pathway in OS cells. Cells apoptosis, reactive oxygen species (ROS) and mitochondrial membrane potential (MMP) were detected using fluorescence staining and flow cytometry. Caspase-3 activity was detected using a commercial kit. The levels of cytoplasmic cytochrome c, mitochondrial cytochrome c, bcl-2, bax, caspase-9, Fas, FasL, caspase-8 and caspase-3 were detected by Western blotting. z-VAD-fmk was applied to block both intrinsic and extrinsic apoptosis pathways, and cells apoptosis was also tested. Furthermore, we used z-LEHD-fmk and Fas blocking antibody to inhibit intrinsic and extrinsic pathways, separately, and the selectivity of oleandrin on these pathways was explored. Results showed that oleandrin induced the apoptosis of OS cells, which was accompanied by an increase in ROS and a decrease in MMP. Furthermore, cytochrome c level was reduced in mitochondria but elevated in the cytoplasm. Caspase-3 activity was enhanced by oleandrin in a concentration- and time-dependent manner. Oleandrin also down-regulated the expression of bcl-2, but up-regulated bax, caspase-9, Fas, FasL, caspase-8 and caspase-3. In addition, the suppression of both apoptotic pathways by z-VAD-fmk greatly reverted the oleandrin-induced apoptosis. Moreover, the suppression of one pathway by a corresponding inhibitor did not affect the regulation of oleandrin on another pathway. Taken together, we concluded that oleandrin induced apoptosis of OS cells via activating both intrinsic and extrinsic apoptotic pathways.

## 1. Introduction

Oleandrin, a polyphenolic component of cardiac glycosides, has been widely reported to play an anti-tumor effect on many types of tumor cells in vitro. Numerous evidences have suggested that oleandrin may become a suitable agent for tumor chemotherapy due to its selective cytotoxicity and radiotherapy/chemotherapy sensitization [1,2,3,4]. A first-in-human in vivo study and a clinical Phase I trial have also reported oleandrin’s superior tolerability and low toxicity in patients with solid tumors [5,6]. The anti-tumor effect of oleandrin in vitro involves in the regulation of diverse molecular biological processes, including the inhibition of the phosphorylation of protein kinase B (Akt) and down-regulation of phosphatidyl inositol 3-kinase (PI3K)/mammalian target of rapamycin (mTOR) pathway [3,7], inhibition of nuclear factor kappa B (NF-κB)/c-Jun NH2-terminal kinase (JNK) signaling pathway [8], activation of death receptor/Apo2L/tumor necrosis factor (TNF)-related apoptosis-inducing ligand (TRAIL) apoptosis signaling pathway [9], suppression of interaction between fibroblast growth factor-2 (FGF-2) and Na^+^, K^+^-ATPase pump [10], induction of intracellular reactive oxygen species production [11], and stimulation of intracellular Ca^2+^ increases and activation of caspase cascade apoptotic signals [12].

Osteosarcoma (OS) is still an intractable bone malignance with high mortality due to frequent recurrence, early metastasis and chemotherapeutic toxicity, even though the improvement of modified surgical techniques and novel chemotherapies has reduced patients’ mortality to a certain extent [13,14]. The heterogeneity and genomic complexity of OS also challenge its chemotherapy and molecular targeted therapy [13]. Hence, it is important to identify a novel agent with selective cytotoxicity against OS cells.

Our previous study has identified the anti-proliferative and anti-invasive effects of oleandrin on OS cells, which indicated the potential value of this compound in OS treatment [15]. Meanwhile, we also observed that oleandrin modulates the expression of certain apoptosis-related genes, including c-myc, cyclin D1 and survivin, which ultimately promotes cells apoptosis through the enhancement of apoptotic signal pathways [15,16,17]. It is well known that two mainly apoptosis pathways, the intrinsic (mitochondria) and extrinsic (death receptor) pathways, are involved in the regulation of tumorigenesis. However, the potential regulation of oleandrin on the two apoptotic pathways in OS cells is still uncertain. For this reason, we focus on exploring this issue in the current study.

## 2. Results

### 2.1. Oleandrin Has a Selectively Antitumor Effect on OS Cells

**Figure 1:**
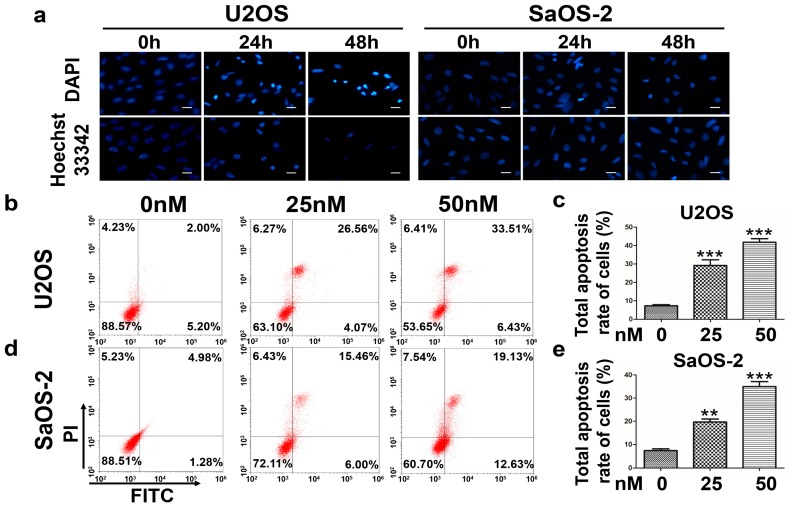
(**a**) DAPI and Hoechst staining of U2OS and SaOS-2 cells treated with different concentrations of oleandrin; Bar scale: 30 μm; (**b**) The apoptosis of U2OS cells treated with different concentrations of oleandrin that detected by flow cytometry (FCM); (**c**) Statistical analysis of the total apoptosis rate of U2OS cells followed by FCM detection; *n* = 3, Mean ± SEM; ** *p* < 0.01, *** *p* < 0.001, vs. control (0 nM) group; (**d**) The apoptosis of SaOS-2 cells treated with different concentrations of oleandrin that detected by FCM; and (**e**) Statistical analysis of the total apoptosis rate of SaOS-2 cells followed by FCM detection; *n* = 3, Mean ± SEM; ** *p* < 0.01, *** *p* < 0.001, vs. control (0 nM) group.

Both 4’,6-diamidino-2-phenylindole (DAPI) and Hoechst 33342 staining indicated that the nuclei of U2OS and SaOS-2 cells presented an uneven morphology with obvious pyknosis or even karyolysis in response to an increased treatment time with oleandrin. These results are typical of cell apoptosis (Figure 1a). In addition, flow cytometry (FCM) analysis showed that oleandrin induced cells apoptosis. After treatment with various concentration of oleandrin (0, 25, 50 nM) for 24 h, the total apoptosis rate of U2OS cells gradually increased, from approximately 7.3% in the control group to about 29.2% in the 25 nM oleandrin-treated group and 41.7% in the 50 nM oleandrin-treated group (Figure 1b). And, the difference was statistically significant (25 vs. 0 nM, *p* < 0.001; 50 vs. 0 nM, *p* < 0.001; Figure 1c). Similarly, the total apoptosis rate of SaOS-2 cells treated with oleandrin was increased along with drug concentration, ranging from approximately 7.4% in the control group to approximately 19.7% in the 25 nM oleandrin-treated group and 34.9% in the 50 nM oleandrin-treated group (Figure 1d). The difference was also statistically significant (25 vs. 0 nM, *p* < 0.01; 50 vs. 0 nM, *p* < 0.001; Figure 1e).

**Figure 2:**
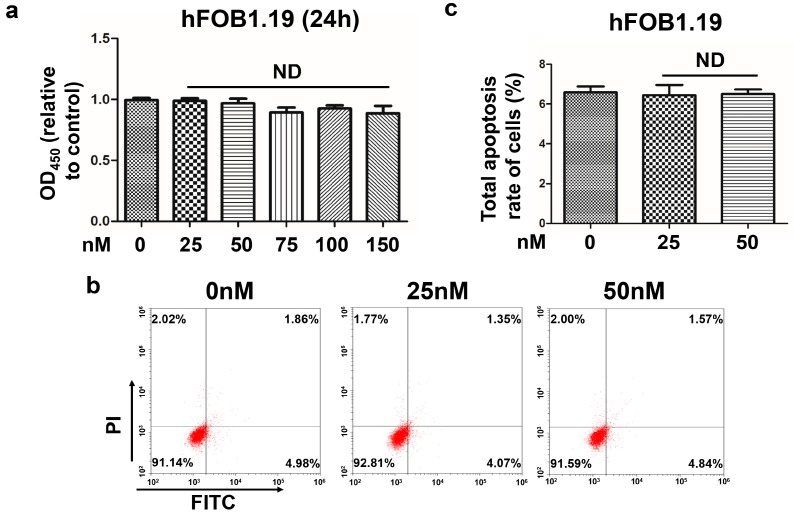
(**a**) The vitality of hFOB1.19 cell treated with different concentrations of oleandrin for 24 h that detected by CCK-8 assays; *n* = 5, Mean ± SEM; ND: no difference, vs. control (0 nM) group; (**b**) The apoptosis of hFOB1.19 cells treated with different concentrations of oleandrin that detected by FCM; and (**c**) Statistical analysis of the total apoptosis rate of hFOB1.19 cells followed by FCM detection; *n* = 3, Mean ± SEM; ND: no difference, vs. control (0 nM) group.

hFOB1.19 cells were treated with various concentration of oleandrin (0, 25, 50, 75, 100 and 150 nM) for 24 h, and cell viability was detected by CCK-8 assay. We observed that the viability was not significantly changed even for concentrations up to 150 nM (Figure 2a), which was well above the effective concentration of oleandrin in OS cells (50 nM). On the other hand, we did not observe any significant decline of the total apoptosis rate of oleandrin-treated hFOB1.19 cells when a concentration of 50 nM was used (Figure 2b,c).

### 2.2. Oleandrin Increases the Reactive Oxygen Species (ROS) in OS Cells

**Figure 3:**
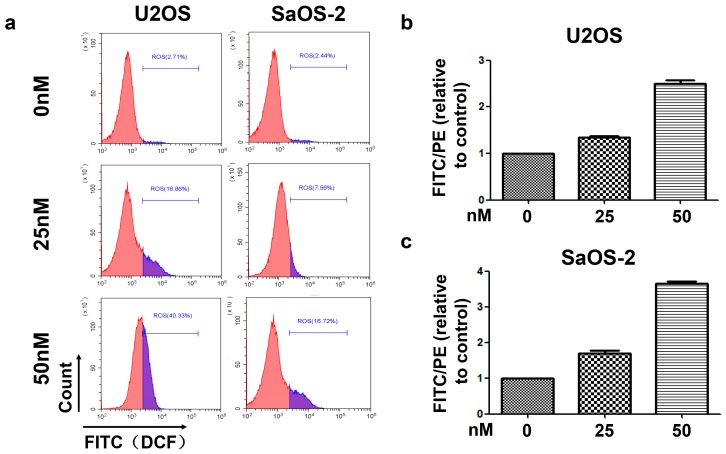
(**a**) Intracellular ROS level of U2OS and SaOS-2 cells treated with different concentrations of oleandrin that detected by FCM (purple region means the percentage of cells with positive DCF); (**b**) Semi-quantitative analysis of relative mitochondrial membrane potential (MMP) level (ratio of FITC/PE) in U2OS cells that detected by FCM, relative to the control (0 nM) group; *n* = 3, Mean ± SEM; and (**c**) Semi-quantitative analysis of relative MMP level (ratio of FITC/PE) in SaOS-2 cells detected by FCM, relative to the control (0 nM) group; *n* = 3, Mean ± SEM.

ROS generation is one of the hallmarks of cell apoptosis. The intracellular ROS level in response to oleandrin treatment was detected using FCM and the percentage (%) of cells with positive 2’,7’-Dichlorofluorescein (DCF), the fluorescence spectrum was similar to fluorescein isothiocyanate (FITC) was used to reflect the level of ROS level. We determined that the percentage of DCF-positive U2OS cells in 25 nM oleandrin-treated group (18.86%) and 50 nM group (40.33%) was higher than that of the 0 nM (control) group (2.71%) (Figure 3a). In keeping with these findings, the percentage of DCF-positive SaOS-2 cells was increased in both groups treated with 25 nM (7.56%) and 50 nM (16.72%) oleandrin compared with controls (2.44%) (Figure 3a). These results indicated that oleandrin induced the production of intracellular ROS in OS cells.

### 2.3. Oleandrin Decreases the Mitochondrial Membrane Potential (MMP) in OS Cells

The intracellular MMP level is determined using FCM and calculated by the ratio of the fluorescence intensity of FITC to the fluorescence intensity of phycoerythrin (PE). The increase of this ratio denotes a decreased MMP level and the occurrence of apoptosis. Our results demonstrated that, compared with the control group, the FITC/PE fluorescence intensity ratio in oleandrin-treated U2OS cells gradually increased about 1.7-fold in 25 nM group and 3.7-fold in 50 nM group (Figure 3b). Likewise, the ratio in oleandrin-treated SaOS-2 cells was also obviously increased approximately 1.3-fold in 25 nM group and 2.5-fold in 50 nM group in comparison with controls (Figure 3c). These results confirmed that the MMP level in oleandrin-treated OS cells was significantly diminished.

### 2.4. Oleandrin Induces Cytochrome C Released from Mitochondria to Cytoplasm

**Figure 4:**
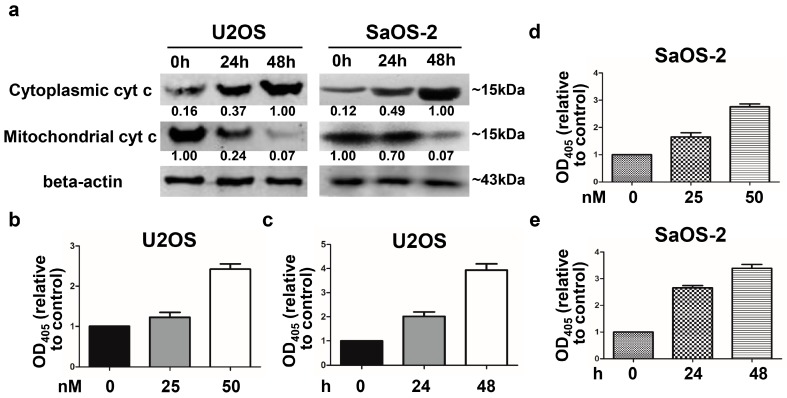
(**a**) Protein expression of cytoplasmic and mitochondrial cytochrome c detected by western blot and the numbers below every picture represent semi-quantitative analysis of each line; (**b**,**c**) Relative OD_405_ value of U2OS cells treated with different concentrations (**b**) and different time points (**c**) of oleandrin, relative to the corresponding controls; *n* = 3, Mean ± SEM; and (**d**,**e**) Relative OD_405_ value of SaOS-2 cells treated with different concentrations (**d**) and different time points (**e**) of oleandrin, relative to the corresponding controls; *n* = 3, Mean ± SEM.

The expression of cytochrome c in oleandrin-treated cells was determined by western blot analysis on separated (distinct) mitochondrial and cytoplasmic protein. The results showed that after treatment with oleandrin for different time periods (0, 24, and 48 h), the expression of mitochondrial cytochrome c in U2OS cells was significantly down-regulated, whereas that of cytoplasmic cytochrome c was notably up-regulated (Figure 4a). Similarly, oleandrin treatment caused the down-regulation of cytochrome c in mitochondria but the up-regulation of cytochrome c in the cytoplasm of SaOS-2 cells (Figure 4a). Thus, we observed that oleandrin treatment induced the release of cytochrome c from the mitochondria to the cytoplasm in OS cells.

### 2.5. Oleandrin Enhances Caspase-3 Activity

Caspase-3 activity in oleandrin-treated U2OS and SaOS-2 cells was determined using a caspase-3 Colorimetric Assay kit and the absorbance at the wavelength of 405 nm was determined. Caspase-3 activity was represented by the relative OD_405_ value in the experiment group to the OD_405_ value in the corresponding control group. The relative OD_405_ value in oleandrin-treated U2OS cells was enhanced about 1.2-fold in the 25 nM group and 2.4-fold in the 50 nM group (Figure 4b); and 2.0-fold in the 24 h group and 3.9-fold in the 48 h group (Figure 4c). Similarly, the relative OD_405_ value in oleandrin-treated SaOS-2 cells also increased about 1.7-fold in the 25 nM group and 2.8-fold in the 50 nM group (Figure 4d); and the 2.7-fold in the 24 h group and 3.4-fold in the 48 h group (Figure 4e). These results demonstrated that oleandrin significantly increased caspase-3 activity in a concentration- and time-dependent manner in both OS cells.

### 2.6. Oleandrin Regulates Apoptosis-Related Proteins in Intrinsic and Extrinsic Apoptosis Signal Pathways

**Figure 5:**
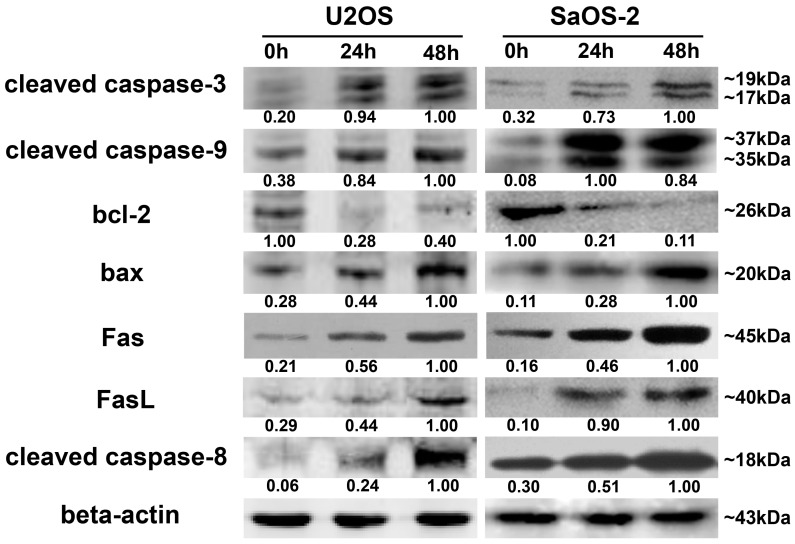
The expression of marker proteins (bcl-2, bax, caspase-9, Fas, FasL, caspase-8 and caspase-3) in the intrinsic and extrinsic apoptotic pathways detected by western blot. The numbers below every picture denote the semi-quantitative analysis of each line.

Both U2OS and SaOS-2 cells were treated with oleandrin for different times (0, 24 and 48 h) and the protein levels of key members of intrinsic and extrinsic apoptotic pathways, including bcl-2, bax, cleaved caspase-9, Fas, FasL, cleaved caspase-8 and cleaved caspase-3 were detected by Western blotting. The results revealed that in both OS cell lines, the levels of bax, cleaved caspase-9, Fas, FasL, cleaved caspase-8 and cleaved caspase-3 were significantly up-regulated by oleandrin after treatment for 48 h. Conversely, the expression of bcl-2 was significantly down-regulated along with treatment time (Figure 5).

### 2.7. The Pro-Apoptotic Effect of Oleandrin Is Dependent on both Intrinsic and Extrinsic Apoptotic Pathways

**Figure 6:**
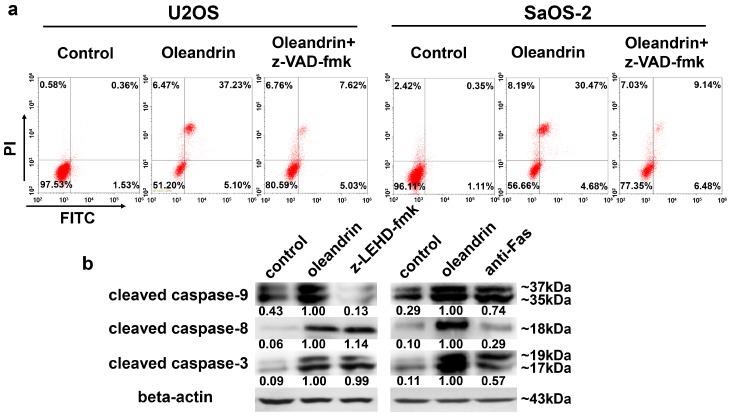
(**a**) Apoptosis changes of cells that pre-treated with z-VAD-fmk followed by oleandrin (50 nM) addition that detected by FCM and (**b**) Protein expression of cleaved caspase-9, cleaved caspase-8 and cleaved caspase-3 in U2OS cells detected by western blot after pre-treating with z-LEHD-fmk or a Fas blocking antibody. The numbers below every picture represent semi-quantitative analysis of each line.

In order to explore whether the pro-apoptotic effect of oleandrin was dependent on intrinsic or extrinsic apoptotic pathways, both U2OS and SaOS-2 OS cells were first treated with a pan-caspase inhibitor named z-VAD-fmk to block both pathways, and then the pro-apoptotic effect of oleandrin was tested. The results showed that the pro-apoptotic effect of oleandrin was mostly restrained by the application of z-VAD-fmk. The result of FCM demonstrated that the total apoptosis rate was lower in the oleandrin combined with z-VAD-fmk group (12.65%) than that of the oleandrin-treated alone group (42.33%) in U2OS cells (Figure 6a). Similarly, the total apoptosis rate was also lower in the oleandrin combined with z-VAD-fmk group (15.62%) than that of oleandrin-treated alone group (35.15%) in SaOS-2 cells (Figure 6a). These results suggested that the pro-apoptotic effect of oleandrin was attenuated by z-VAD-fmk. Hence, both intrinsic and extrinsic apoptotic pathways might be involved in the pro-apoptotic process of oleandrin.

Furthermore, we used a caspase-9 inhibitor (z-LEHD-fmk) and a Fas blocking antibody to interrupt the intrinsic and extrinsic apoptotic pathways in U2OS cells, separately. Then, the selective effect of oleandrin on the two apoptotic pathways was analyzed. The results obtained by blocking the intrinsic apoptotic pathway (with z-LEHD-fmk) showed that cleaved caspase-9 was notably decreased, which suggested a successful suppression of intrinsic apoptotic pathway. However, the expression of cleaved caspase-3 was still increased and accompanied by the up-regulation of cleaved caspase-8, in comparison with the control group. These data revealed that oleandrin activated the extrinsic apoptotic pathway independently from the intrinsic pathway (Figure 6b). In addition, the application of Fas blocking antibody caused the decrease of cleaved caspase-8, which indicated the successful inhibition of the extrinsic apoptotic pathway. Nevertheless, compared with the control group, the expression of cleaved caspase-3 was also increased and accompanied by the up-regulation of cleaved caspase-9 (Figure 6b). These data suggested that oleandrin could also induce the activation of intrinsic apoptosis pathway in OS cells that are independent of the extrinsic pathway. Taken together, all the results demonstrated that both intrinsic and extrinsic apoptosis pathways were activated by oleandrin in inducing the apoptosis of OS cells.

## 3. Discussion

Our previous study determined that oleandrin has an antitumor effect on OS cells and regulates the expression of certain apoptosis-related genes [15]. Current evidence also demonstrated that oleandrin induced apoptosis in both U2OS and SaOS-2 cells; in fact, they presented a typical apoptotic morphology with obvious nuclei pyknosis or karyolysis, and showed a remarkable increase of the total apoptosis rate. Furthermore, the induction of apoptosis by oleandrin had a selective effect. In fact, it did not suppress the viability of normal hFOB1.19 osteoblast cells, significantly inducing their apoptosis. Even if many studies have reported the pro-apoptotic function of oleandrin in other human tumors through the regulation of apoptosis-associated signaling pathways [1,4,11,18], the potential apoptotic mechanism regulated by oleandrin in OS is not well-known. It is well known that two main apoptotic pathways, including the intrinsic (mitochondria) and extrinsic (death receptor) pathways are involved in the regulation of tumorigenesis [19]. Thus, in this study, we mainly explored the regulating effect of oleandrin on these pathways.

In the intrinsic apoptotic pathway, B-cell lymphoma 2 (bcl-2) family proteins, which include the anti-apoptotic proteins bcl-2 and bcl-xl, as well as the pro-apoptotic proteins bax, bak, bad and bim, are primary up-stream molecules that respond to apoptosis signals. In fact, a decrease in the bax/bcl-2 ratio is commonly observed in various tumors, and closely correlates with the inhibition of cell apoptosis, insusceptibility to undergo apoptosis, tumor recurrence and patients’ poor prognosis [20,21]. These proteins are mostly localized in the mitochondrial membrane and are involved in the regulation of the mitochondrial membrane function. Mitochondria are the crucial production sites of ROS that regulate the intracellular redox status. Physiologically, a low concentration of ROS can be used as a second messenger to participate in multiple signaling pathways that maintain homeostasis. When cells are stimulated by apoptosis signals, the aforementioned apoptosis-associated proteins rapidly react to the stress and dramatically induce ROS generation [22]. Meanwhile, a high concentration of ROS in mitochondria may reduce the MMP that enhances the mitochondrial permeability, and induce certain mitochondrial factors (such as cytochrome c) released from the mitochondria to the cytoplasm [22,23]. In the cytoplasm, cytochrome c is involved in the formation of cytochrome c/Apaf-1/caspase-9-containing apoptosome, which subsequently results in the activation of caspase-9 and caspases-3 [23]. Caspases, the cytoplasmic aspartate-specific cysteinic proteases, play a central role in cell apoptosis by transferring apoptotic signals. The activation of their cascade is the hallmark of apoptosis [24]. Activation of caspase-3, the last step of the cascade, leads to the degradation of specific cellular substrates and induces cell apoptosis.

Our results showed that oleandrin significantly up-regulated the expression of bax but down-regulated the level of bcl-2 with the increase of treating time, which implied an increase of bax/bcl-2 ratio in oleandrin-treated OS cells. In addition, the intracellular ROS level of both OS cells treated with oleandrin was substantially elevated with the increase of drug concentration. However, the intracellular MMP level presented a decreasing trend, as shown by the fact that the ratio of the fluorescence intensity of FITC to the fluorescence intensity of PE was increased after treatment with various concentrations of oleandrin. Furthermore, we determined the levels of cytoplasmic and mitochondrial cytochrome c by separating the cytoplasmic and mitochondrial proteins. The results demonstrated that oleandrin treatment resulted in a strong down-regulation of the mitochondrial cytochrome c, but a strong up-regulation of the cytoplasmic cytochrome c, which suggested that oleandrin could induce the cytochrome c released from the mitochondria to the cytoplasm. In addition, oleandrin also caused the increased expression of cleaved caspase-9 and cleaved caspase-3, and simultaneously enhanced the caspase-3 activity in a time- and concentration-dependent fashion. These findings revealed that oleandrin initiated the activation of the mitochondria-dependent apoptotic pathway in both U2OS and SaOS-2 cells through up-regulation of the pro-apoptotic protein bax and down-regulation of the anti-apoptotic protein bcl-2. Subsequently, the extensive generation of intracellular ROS and the reduction of MMP level were induced by oleandrin, which led to the release of cytochrome c from the mitochondria to the cytoplasm. As a result, the caspase cascade reaction, including the activation of caspase-9 and caspase-3, was initiated, ultimately leading to the occurrence of cells apoptosis.

In the extrinsic apoptotic pathway, the death-inducing signaling complex (DISC), which include the TNF receptor, TRAIL receptors, Fas and FasL, can be triggered by apoptotic signals and drive the activation of caspase-8 [25]. Activated caspase-8 can transduce apoptotic signals downstream from the pathway by directly cleaving caspase-3 or indirectly cleaving Bid to tBid. As a result, tBid further translocates to the mitochondrial membranes and induce its permeabilization [25,26]. These events eventually cause the occurrence of apoptosis as well. Likewise, we also determined that the expression of Fas, FasL and cleaved caspase-8 were all significantly increased after incubating with oleandrin for different times. These results demonstrated that oleandrin also prompted the activation of the death receptor apoptotic pathway by up-regulating the expression of the death receptor Fas and its ligand FasL, and inducing the caspase cascade reaction with the activation of caspase-8.

Furthermore, z-VAD-fmk, a pan-caspase inhibitor that is permeable through the cellular membrane, was applied to test whether oleandrin-induced apoptosis was dependent either on the intrinsic or extrinsic pathway. z-VAD-fmk induces caspases-3 alkylation and blocks its activity, which causes the inactivation of the caspase cascade reaction both in intrinsic and extrinsic apoptotic pathways [27]. The result of FCM demonstrated that the total apoptosis rate of both OS cell lines pretreated with the z-VAD-fmk was not only significantly lower than that of the group treated with oleandrin, but also nearly reverted to the normal level. These findings illustrated the application of z-VAD-fmk attenuated cells apoptosis that was induced by oleandrin and suggested that oleandrin-induced apoptosis may be associated with the caspases cascade activation in both intrinsic and extrinsic apoptotic pathways.

In order to analyze the selective effect of oleandrin on intrinsic and extrinsic apoptotic pathways, a caspase-9 specific inhibitor (z-LEHD-fmk) and a Fas blocking antibody were used to separate interrupt these pathways in U2OS cells, followed by the determination of pathways’ activity. We observed that, although the intrinsic apoptotic pathway was successfully suppressed in cells that pretreated with z-LEHD-fmk on the basis of the decreased cleaved caspase-9, the expression of cleaved caspase-8 and cleaved caspase-3 in these cells was still increased with the treatment of oleandrin. These findings revealed that the activation of oleandrin on the extrinsic apoptotic pathway in OS cells was independent of the intrinsic apoptotic pathway. In addition, although the inhibition of the extrinsic apoptotic pathway occurred in cells that are pretreated with a Fas blocking antibody on the basis of the decreased cleaved caspase-8, the expression of cleaved caspase-9 and cleaved caspase-3 was still increased in these cells. These findings also suggested that the activation of oleandrin on the intrinsic apoptotic pathway in OS cells was independent of the extrinsic apoptotic pathway. Taken together, these results indicated that both intrinsic and extrinsic apoptotic pathways were involved in the oleandrin-mediated apoptosis.

Meanwhile, we also found that normal hFOB1.19 osteoblast cells used in this study were insusceptible to oleandrin treatment. This finding was similar to the results of previous studies. Sreenivasan Y et al. [4] revealed that oleandrin could selectively induce apoptosis of human malignant cells, but not normal primary cells such as peripheral blood mononuclear cells (PBMC) or neutrophils. In addition, Calderón-Montaño JM et al. [28] also found that an extract from *Nerium oleander* selectively induced the death of lung cancer cells but not nonmalignant lung fibroblasts. An explanation of the potential mechanism of oleandrin-mediated apoptosis may be that normal cells lack an elevated expression of certain subunits of Na^+^, K^+^-ATPase (such as α3), which is typical of tumor cells and positively correlates with the sensitivity to oleandrin. An additional possible cause may be that the compounds derived from cardiac glycosides exert an intense inhibition of glycolysis in tumor cells, which have a stronger reliance on glycolysis for their survival than normal cells. However, the selective effect of oleandrin on promoting apoptosis in OS cells but not normal osteoblast cells is uncertain and therefore warrants further investigation.

## 4. Materials and Methods

### 4.1. Drug Preparation

Oleandrin was obtained from Sigma-Aldrich Chemical Co. (St. Louis, MO, USA). The molecular structure of oleandrin is showed in the Supplementary file (Figure S1). The purity was approximately 99%, as analyzed by HPLC. A 1 mmol/L stock solution (Mr = 576.73) was prepared by dissolving oleandrin in 100% DMSO (Sigma-Aldrich) and was stored at −80 °C. All subsequent dilutions were made in the medium. The final concentration of DMSO was confirmed to be less than 0.1%. The pan-caspase inhibitor with cell membrane permeability, z-VAD-fmk, was purchased from Beyotime (Beyotime Biotech, Nanjing, China). The caspase-9 inhibitor, z-LEHD-fmk, was obtained from Sigma (Sigma-Aldrich Chemical Co.). The Fas blocking antibody (Abcam, Cambridge, MA, USA) was kindly donated by Wei Zhang from the Institute of Zoology, Chinese Academy of Science.

### 4.2. Cell Lines and Cell Culture

Two human OS cell lines, U2OS and SaOS-2 were kindly donated by Medical Research Center of Peking University Third Hospital and derived from the China Infrastructure of Cell Line Resources. The human normal osteoblastic cell line hFOB1.19 used in this study was previously preserved by our research team. OS cells were cultured in McCoy’s 5A medium (HyClone, Logan, UT, USA) containing 1% penicillin-streptomycin (10,000 U/mL) (Gibco, Grand Island, NY, USA) and 10% fetal bovine serum (FBS) (Gibco), and maintained at 37 °C in a humidified 5% CO_2_ incubator. hFOB1.19 cells were cultured in a 1:1 mixture of Ham’s F12 Dulbecco’s Modified Eagle’s Medium (DMEM), with 2.5 mM l-glutamine. The medium was supplemented with 10% fetal bovine serum and G418 (0.3 mg/mL, Gibco, Carlsbad, CA, USA), and maintained in a humidified atmosphere with 5% CO_2_ at 34 °C.

### 4.3. DAPI and Hoechst 33342 Staining

Cells were incubated in 24-well plates and treated with 50 nM oleandrin for various time periods (0, 24 and 48 h). DAPI staining (Beyotime Biotechnology, Nanjing, China) was applied to 4% paraformaldehyde fixed cells following membrane perforation with Triton X-100. Hoechst 33342 staining (Beyotime Biotechnology, Nanjing, China) was performed diretly on live cells according to the protocol. The morphological of cell nuclei was observed using a fluorescence microscope (Leica DM3000, Frankfurt, Germany).

### 4.4. Annexin V-FITC/PI Apoptosis Assay

Cell apoptosis was detected using an Annexin V-FITC/PI apoptosis detection kit (BioVision, San Francisco, CA, USA) according to the manufacturer’s protocol. Briefly, cells were seeded into 6-well plates and incubated with different concentration of oleandrin (0, 25 and 50 nM) for 24 h. After that, the cells were collected and stained with Annexin V-FITC and PI. Then, they were immediately acquired with a CytoFLEX flow cytometer (Beckman Coulter, Brea, CA, USA) and their fluorescence intensity was detected.

### 4.5. Intracellular ROS Assay

The intracellular ROS level was detected using a Reactive Oxygen Species Assay Kit (Beyotime Biotechnology, Beijing, China). Briefly, after treatment with various concentrations of oleandrin, the cells were washed with PBS and incubated for 30 min in the incubator with 100 µL PBS containing 10 µm DCFH-DA, according to the protocol. DCFH-DA is a non-fluorescent cell permeable agent that can be hydrolyzed to DCFH by intracellular esterases. Moreover, DCFH is a cell impermeable agent that can be oxidized into DCF by intracellular ROS. Additionally, DCF is an element with a fluorescence spectrum similar to that of FITC and that can be detected by FCM, reflecting the level of intracellular ROS.

### 4.6. Mitochondrial Membrane Potential (MMP) Assay

Changes in intracellular MMP after oleandrin treatment were determined with a commercial Mitochondrial Membrane Potential Assay Kit with JC-1 (Beyotime Biotechnology, Beijing, China) according to the protocol. Briefly, the cells were treated with diverse concentration of oleandrin (0, 25 and 50 nM) for 24 h, incubated with 1 mL JC-1 dye for 20 min at 37 °C in the dark, and then washed twice with 1 × JC-1 staining buffer. In healthy cells, JC-1 selectively accumulates in the intact mitochondria to form multimer J-aggregates that emit at 590 nm (PE, red fluorescence). However, in apoptotic cells, JC-1 enters the cytoplasm as a monomer, emitting at 527 nm (FITC, green fluorescence). The ratio obtained by dividing the fluorescence intensity of FITC by the fluorescence intensity of PE negatively correlates with the MMP level. As apoptotic cells present a high intensity of FITC and a low intensity of PE, so the increase of this ratio indicates a decreased MMP level and the occurrence of apoptosis. The result was detected and analyzed using FCM. The experiment was performed in triplicate for each group.

### 4.7. Caspase-3 Activity Assay

Oleandrin-treated cells were digested with trypsin and thoroughly lysed on ice for 30 min accompanied by vortexing every 10 min. The concentration of protein was detected using a BCA Protein Assay Kit according to the protocol (Applygen Technologies Inc., Beijing, China). A total of 150 μg proteins were detected using a Caspase-3 Colorimetric Assay kit (Beyotime Biotechnology, Beijing, China) and the absorbance at 405 nm was determined using an Thermo Scientific^TM^ automatic ELIASA microplate reader (Thermo Fisher Scientific Inc., Waltham, MA, USA). The caspase-3 activity was represented by the relative OD_405_ value in the experiment group to the OD_405_ value in the corresponding control group. The increase of the relative OD_405_ means the enhancement of the caspase-3 activity. The assay was performed in triplicate for each group.

### 4.8. CCK-8 Assay

hFOB1.19 cells were seeded in a 96-well dish at a final density of 6 × 10^3^ cells/well and incubated overnight. Then, cells were treated with various concentration of oleandrin (0, 25, 50, 75 and 100 nM) for 24 h. Then, CCK-8 agent (Dojindo Laboratories, Kumamoto, Japan) was added to each well and incubated for another 3 h. The absorption at 450 nm was determined using an automatic ELIASA microplate reader as reported above. Five replicate wells were used for each treatment.

### 4.9. Western Blot (WB)

Cells were treated with 50 nM oleandrin for different time (0, 24 and 48 h). At each time point, total protein lysate from each group were isolated using a Total Protein Extraction Kit (Applygen Technologies Inc., Beijing, China), and the cytoplasmic and mitochondrial proteins were separated using a Cytoplasmic and Mitochondrial Protein Extraction Kit (Beyotime Biotechnology, Beijing, China). Protein concentration was determined as described above. The same amount of proteins was resolved on SDS-PAGE and transferred to a nitrocellulose membrane. The blots were blocked with 5% BSA at room temperature for 1 h and incubated with the following primary antibodies purchased from Cell Signaling Technology (CST, Beverly, MA, USA): bcl-2 rabbit monoclonal antibody (1:1000), bax rabbit monoclonal antibody (1:1000), caspase-3 rabbit monoclonal antibody (1:1000), caspase-9 rabbit polyclonal antibody (1:1000), Fas rabbit monoclonal antibody (1:1000), FasL rabbit polyclonal antibody (1:1000) and caspase-8 mouse monoclonal antibody (1:1000) as well as β-actin mouse monoclonal antibody (1:3000, CWBIO, Beijing, China). Cytochrome c in cytoplasm and mitochondria were determined with cytochrome c rabbit polyclonal antibody (1:500, Santa Cruz Biotechnology, Santa Cruz, CA, USA). IRDye 800CW conjugated goat (polyclonal) anti-rabbit and anti-mouse IgG secondary antibody (1:10,000 dilution) (LI-COR^®^ Biosciences, Lincoln, NE, USA) were used as secondary antibodies. The fluorescent blots were analyzed with an Odyssey CLx infrared imaging system (LI-COR^®^ Biosciences), and the gray values were measured using the Odyssey V3.0 software (LI-COR^®^ Biosciences). The expression of all proteins was quantified with respect to the expression of β-actin.

### 4.10. Statistical Analysis

All data were analyzed with the IBM SPSS statistics 20.0 software (IBM, Armonk, NY, USA) and represented as the mean ± standard error of the mean (SEM). For the comparisons with the control group, statistical analyses were performed using one-way analysis of variance (ANOVA) with post hoc Dunnet analysis. Pairwise comparison was applied using ANOVA with post hoc Tukey’s test. A value of *p* < 0.05 was considered to be statistically significant.

## 5. Conclusions

Based on the results described above, we can conclude that oleandrin induces the apoptosis of OS cells via the activation of intrinsic- and extrinsic-dependent pathways, and also induces the caspases cascade in both apoptotic pathways.

## Supplementary Materials

Supplementary materials can be found at www.mdpi.com/1422-0067/17/11/1950/s1.

## Abbreviations

OS	Osteosarcoma
MMP	Mitochondrial membrane potential
ROS	Reactive oxygen species
Caspase	Cysteinyl aspartate specific proteinase
FCM	Flow cytometry
FITC	Fluorescein isothiocyanate
FBS	Fetal bovine serum
Akt	Protein kinase B
PI3K	Phosphatidyl inositol 3-kinase
mTOR	Mammalian target of rapamycin
NF-κB	Nuclear factor kappa B
JNK	c-Jun NH2-terminal kinase
TNF	Tumor necrosis factor
TRAIL	TNF-related apoptosis-inducing ligand
Bcl-2	B-cell lymphoma 2
DISC	Death-inducing signaling complex
FGF-2	Fibroblast growth factor-2
PBMC	peripheral blood mononuclear cells
DMEM	Dulbecco’s Modified Eagle’s Medium
DCF	2’,7’-Dichlorofluorescein
DAPI	4’,6-diamidino-2-phenylindole
FITC	Fluorescein isothiocyanate
PE	Phycoerythrin
