# Supplementary material for: Regulation of Intrinsic and Extrinsic Apoptotic Pathways in Osteosarcoma Cells Following Oleandrin Treatment
Source: Int J Mol Sci. 2016 Nov 23;17(11):1950. doi: 10.3390/ijms17111950 (PMC5133944; doi:10.3390/ijms17111950)
Supplement: Supplementary file 1 [file ijms-17-01950-s001.pdf]

Supplementary Materials: Regulation of Intrinsic and Extrinsic Apoptotic Pathways in Osteosarcoma Cells Following Oleandrin Treatment

Yunlong Ma, Bin Zhu, Lei Yong, Chunyu Song, Xiao Liu, Huilei Yu, Peng Wang, Zhongjun Liu and Xiaoguang Liu

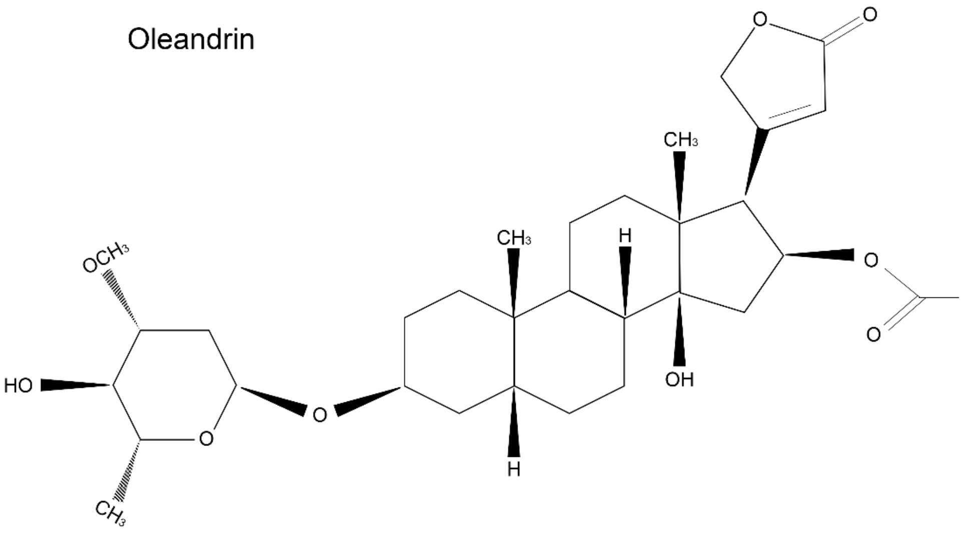

Figure S1. Molecular Structure of Oleandrin [1,2].

References

1. Kumar, A.; de, T.; Mishra, A.; Mishra, A.K. Oleandrin: A cardiac glycosides with potent cytotoxicity. *Pharm. Rev.* **2013**, *7*, 131–139.
2. Ni, D.; Madden, T.L.; Johansen, M.; Felix, E.; Ho, D.H.; Newman, R.A. Murine pharmacokinetics and metabolism of oleandrin, a cytotoxic component of *Nerium oleander*. *J. Exp. Ther. Oncol.* **2002**, *2*, 278–285.
